# Sound Visualization Demonstrates Velopharyngeal Coupling and Complex Spectral Variability in Asian Elephants

**DOI:** 10.3390/ani12162119

**Published:** 2022-08-18

**Authors:** Veronika C. Beeck, Gunnar Heilmann, Michael Kerscher, Angela S. Stoeger

**Affiliations:** 1 Department of Behavioural and Cognitive Biology, University of Vienna, 1030 Vienna, Austria; 2 Mecalc Technologies GmbH, 12487 Berlin, Germany

**Keywords:** elephant, vocal communication, source-filter theory, sound production, formant, vocal tract, vocal complexity, graded repertoire, functional morphology, vocalization

## Abstract

**Simple Summary:**

Whether a vocalization is emitted though the mouth or nose impacts on its acoustic shape through specific vocal tract resonances. In human language, nasalized compared to oral vowels can change the meaning of words. African elephants mainly use low-frequency rumbles to communicate and can utter them either through the mouth or trunk. In this study, we used an acoustic camera to visualize the sound emission of rumbles in Asian elephants, which have been studied considerably less than African elephants. We recorded nine captive female Asian elephants and analyzed the acoustic structure of 203 calls. We found that most rumbles (64%) were emitted through the trunk, 21% through the mouth, and surprisingly, 13% simultaneously through the mouth and trunk. Some of the rumbles were combined with orally emitted roars. The nasal rumbles concentrated most spectral energy in lower frequencies, whereas the oral and mixed rumbles showed a broader spectral energy distribution and concentration in higher frequencies, and were louder. The roars were the loudest, broadest and highest in frequency. This study is the first to demonstrate coupled oral and nasal emission in a non–human animal, thereby setting an important framework to study the functions of this acoustic variability in elephant communication and the evolution of vocal flexibility across species.

**Abstract:**

Sound production mechanisms set the parameter space available for transmitting biologically relevant information in vocal signals. Low–frequency rumbles play a crucial role in coordinating social interactions in elephants’ complex fission–fusion societies. By emitting rumbles through either the oral or the three-times longer nasal vocal tract, African elephants alter their spectral shape significantly. In this study, we used an acoustic camera to visualize the sound emission of rumbles in Asian elephants, which have received far less research attention than African elephants. We recorded nine adult captive females and analyzed the spectral parameters of 203 calls, including vocal tract resonances (formants). We found that the majority of rumbles (64%) were nasally emitted, 21% orally, and 13% simultaneously through the mouth and trunk, demonstrating velopharyngeal coupling. Some of the rumbles were combined with orally emitted roars. The nasal rumbles concentrated most spectral energy in lower frequencies exhibiting two formants, whereas the oral and mixed rumbles contained higher formants, higher spectral energy concentrations and were louder. The roars were the loudest, highest and broadest in frequency. This study is the first to demonstrate velopharyngeal coupling in a non-human animal. Our findings provide a foundation for future research into the adaptive functions of the elephant acoustic variability for information coding, localizability or sound transmission, as well as vocal flexibility across species.

## 1. Introduction

In mammals, the length and shape of the vocal tract modulates the sound produced by the vibration of the vocal folds, and thus the information it conveys to the listener [1,2]. Elephants have evolved the longest and one of the most sophisticated vocal tract elongations in mammals, the trunk [3]. Hence, this flexible muscular hydrostat plays a crucial role in their acoustic communication, apart from serving as a multipurpose tool for manipulating food and objects [3,4,5], for sensory information and for tactile, chemical, and gestural communication [6,7,8,9]. The three recent elephant species—the African savannah elephant (*Loxodonta africana*, henceforth African elephant), African forest elephant (*Loxodonta cyclotis*) and Asian elephant (*Elephas maximus*)—share 8 to 10 call types that appear to constitute a rather limited vocal repertoire at first sight but actually exhibits an impressive flexibility in within-call–type variation and the combinations of trunk and laryngeal sounds [10,11]. In addition, single captive individuals of African elephants and one Asian elephant proved capable of adding novel sounds to their repertoire through vocal production learning, a capability whose functional value in their interspecific communication remains unknown [12,13]. The trunk is used not only to produce the iconic high-frequency trumpet, where the exact sound source is not yet localized, but also squeaks, idiosyncratic high-frequency sounds, throbbing sounds, snorts and blows (Beeck, unpublished data, [14,15,16]). By vibrating their massive vocal folds, the elephants produce tonal rumbles [17] with fundamental frequencies reaching into the infrasonic range (<20 Hz), but with energy present in higher harmonics up to about 1000 Hz [18,19]. The rumble is the most common call type used across elephant species for interspecific communication in their complex fission–fusion societies [20,21,22]. The trunk’s resonant properties have been shown to affect the spectral structure of the rumbles in African elephants [23], but this has not, thus far, been studied in Asian elephants. A better understanding of the adaptive values of the vocal flexibility for functional information-coding across the elephant species requires a better understanding of the relationship between the elephant’s unique morphology and its resulting acoustic properties.

### 1.1. The Function of the Vocal Tract in Mammals

Source– and filter–related parameters can be modified independently [1,2]. First, the pulmonary air-flow powers the self–sustaining vibration of the vocal folds, which, when regular, translates to the fundamental frequency (F0) and the harmonic overtones of the call. Then, the vocal tract’s filtering function dampens or enhances the specific frequencies, resulting in acoustic energy peaks across harmonics, i.e., formants. The length and shape of the vocal tract determines the location and amplitude of the formants [24,25,26]. In humans, the low position of the larynx and the flexible movements of the supra-laryngeal articulators (e.g., pharynx, tongue, velum, lips and jaw) dynamically shape the vocal tract and enable us to produce consonants and vowels, the latter being determined by the relative position of the lower three formants [24]. The rapid combination of these phonetic elements allow for the almost unlimited amount of information encoded in human speech. The position of the velum determines whether the vocalization is uttered orally, nasally or through a combination of both of the pathways [27]. The latter, referred to as velopharyngeal coupling, is known in human language as the nasalization of vowels, such as in the French or Hindi language [24,28,29]. In 30% of human languages (WALS dataset), the nasalization of vowels can change the meaning of a word, for example in French *beau* (bo) means “beautiful” and *bon* (bõ) means “good” [28]. Non–human mammals are generally thought to have less control over the shape and dimension of their vocal tracts than humans [26,30,31].

Orally and nasally coupled vocalizations have, to our knowledge, not been conclusively demonstrated in any non–human mammal. They have been suggested based on the non-uniform formant spacings in rhesus macaques (*Macaca mulatta*) [32], Diana monkeys (*Cercopithecus diana*) [33], elephant seals (*Mirounga leonina*) [34] and fallow deer (*Dama dama*) [35]. However, velopharyngeal coupling and vocal tract modifications may have similar effects on the spectral structure of the vocal output [28,29,36]. This means that the velopharyngeal coupling cannot be inferred from the acoustic data alone without evidence of sound emission or velum aperture.

Orally or nasally emitted calls, in contrast, have been demonstrated in numerous species. Here, the formant positions are predicted by the vocal tract length, assuming the vocal tract resembles a uniform tube, where the longer the vocal tract, the lower the formant frequencies and the smaller the spacing between them [1,37,38]. The nasal vocal tract is usually longer than the oral one and the formant positions differ accordingly between the oral and nasal calls. This has been shown in goitered gazelles (*Gazella subgutturosa*) [39], Saiga antelopes (*Saiga tatarica*) [40], red deer (*Cervus elaphus*) [41], sheep (*Ovis aries*) [42] and several lemur species (*Eulemur*) [43], where the nasal and oral call emission was observed through the mouth openings. The oral or nasal call production were further demonstrated using x-ray videos in dogs (*Canis familiaris*), goats (*Capra hircus*), a piglet (*Sus scrofa*) and cotton-top tamarins (*Saguinus oedipus*) [27], and an acoustic camera that visualizes sound emission in African elephants [23].

The formant positions have mainly been shown to code “static” information about the stable long-term attributes of the caller (age, sex, gender) in the context of sexual selection or male–male competition [26,44]. Because of the negative relationship between body size and formant dispersion, some mammals extend their vocal tract to exaggerate their size [45]. The oral vocal tract can be enlarged through lip protrusion (e.g., rhesus macaques [37,46], black and white colobus monkeys (*Colobus guereza*)) or retraction of the larynx (e.g., red deer [47]). The permanent nasal vocal tract elongations, which are more prominent in males, are seen in the proboscic noses of proboscis monkeys [48] and the Saiga antelope’s and elephant seal’s enlarged nasal vestibulums [34,49]. Beyond the mating context, very few studies have examined the variation in the formant patterns caused by articulator modulations in relation to “dynamic” information (arousal, valence, emotion, motivation) [50] or referential signaling [44], except for the studies on the alarm calls of Diana monkeys [33], meerkats (*Suricata suricatta*) [51] and African elephants [52]. These findings challenge the view that the reduced articulatory capacities of non–human mammals limit the potential information coding in formant patterns [31], and call for a broader consideration of the relative spectral contribution of oral and nasal resonances.

### 1.2. Call Emission and Information Coding in Elephant Laryngeal Calls

**Figure 1:**
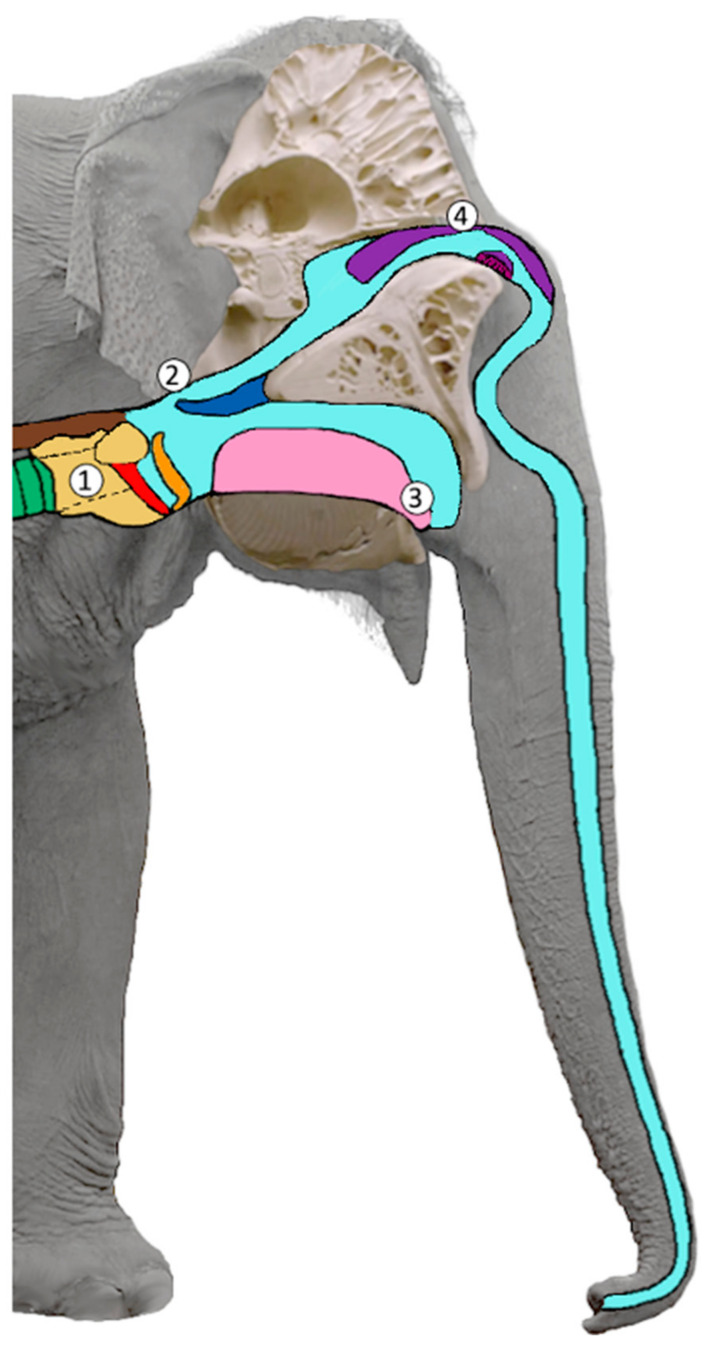
Schematic figure of the vocal tract in sagittal view: (1) larynx (yellow), vocal folds (red), epiglottis (orange), trachea (green), esophagus (brown); (2) velum (dark blue); (3) tongue (pink); (4) nasal cartilages (violet). In this figure, the epiglottis is slightly open unifying the oral and nasal vocal tract. When the (2) velum (dark blue) is lowered and touches the epiglottis (orange) the oral vocal tract can be closed, and the nasal vocal tract opened for purely nasal sound emission (adapted from [67]).

In elephants, the trunk in both of the sexes extremely elongates the nasal vocal tract (up to 2.5 m in African elephant adult females) in relation to the oral path (0.7 to 1 m) (Figure 1) [19]. The rumbles that were uttered through the trunk in three subadult female African elephants exhibited a three-fold reduction in nasal formant positions and spacing (mean F1 = 40 Hz; mean F2 = 169 Hz) as compared to the orally uttered rumbles (mean F1 = 129 Hz; mean F2 = 415 Hz) [23]. During arousal, rumbles often occur in combination with loud roars with a F0 of approximately 200 Hz (in Asian elephants in this current study) [11]. The roars are suggested to be produced laryngeally and the acoustic camera recordings revealed that the African elephants emit their roars orally [16]. In African elephants, rumbles were found to vary gradually [6,53] (but see [54,55]), but to encode perceptional discrete information [52,56,57]. Here, the formant configurations play an important role. They reflect the pronounced sexual dimorphism in the vocal tract dimensions [58], and age and maturity within males [59]. Furthermore, formants vary with individual identity [56], reproductive state [60], dominance rank [61], alarming triggers [52] and also, in calves, the behavioral context [62]. However, the relationship between the morphology of the vocal tract, the sound space and range, and the information encoded is not well understood.

Compared to this extensive body of research on African elephants, the Asian elephant acoustic communication has been considerably less investigated [63,64,65]. Their low-frequency calls were found to vary among individuals (but the formants were not analyzed) [65], and among behavioral contexts, although here individual differences were not considered [66]. DeSilva, 2010, distinguished between growls, which have no spectral energy over 500 Hz and are emitted through a nearly closed mouth, and rumbles, which have a broader frequency spectrum and are uttered through the open mouth. These observations indicate that the Asian elephants, too, emit laryngeal calls through oral or nasal emission.

In the current study, we investigated the sound emission and structure of the rumbles and roars of captive adult female Asian elephants in detail (adult males are more difficult to handle and thus rarer in captivity). We used an acoustic camera array to visualize the sound emission and to measure the sound intensity. By analyzing multiple time and frequency frames per call, we explored the sequential or simultaneous call emission through the oral or vocal tract. We compared the acoustic structure of the call types, estimated the corresponding vocal tract lengths and tested the call–type classification. We discuss commonalities among the mammal vocal tract resonances and the potential function of our findings within the Asian elephant’s vocal communication.

## 2. Materials and Methods

### 2.1. Study Subjects and Housing

The subjects in this study were 9 of the 12 captive adult female Asian elephants located at Tiger Tops Elephant Camp within the buffer zone of the Chitwan National Park, Nepal (coordinates 27.52935, 84.35419). They ranged in age from about 27 to 60 years and shoulder height from 2.40 to 2.55 m (Table 1). The elephants were kept chain-free in corrals of around 0.25 to 1.55 ha in size, in groups of two and one group of three based on social affiliations. The animals were taken on daily trips by their handlers to bath in the nearby Narayani River and to browse in the adjacent grassland or in the rainforest of the buffer zone. All of the elephants were trained and habituated to the presence of humans.

### 2.2. Acoustic Camera and Audio and Video Recordings

We conducted acoustic camera recordings on four days from 9 until 12 of October 2018 at varying times between 6:00 a.m. and 8:00 p.m. The acoustic camera (Star48 AC Pro, gfai Tech GmbH, Berlin, Germany) consists of an array of 48 microphones arranged on a three–armed star around a central camera, for concomitant video and audio recordings. The array is conically tilted forward in the direction of the sound source, creating a back-field suppression of approximately 15 dB. The array was placed about 6–8 m from the vocalizing elephant and connected to a recorder and laptop with the operating program Noise Image (4.10.0). Given the amount of data to be processed, the acoustic camera recordings cannot be conducted continuously, but a pre-trigger and the total recording time (max 360 s) were set before each recording. For the simultaneous and continuous acoustic recordings during the sessions, we used an omni-directional Neumann KM183 condenser microphone that was modified for recording frequencies below 20 Hz (flat-recording down to 5 Hz frequency response: 5 Hz–20 kHz) with a Rycote windshield, which was connected to a Sound Devices 633 (at 48 kHz sampling rate and 16–bit). We used a Sony Camera FD53 for video recordings. The acoustic recordings were stored as .wav files.

### 2.3. Recording Context

Since we were primarily interested in the sound production of the calls before their acoustic variation relevant to the behavioral context, we aimed at recording as many vocalizations as possible, rather than balancing the observational settings across the individuals. We established contexts in which the individual elephants can be stimulated to vocalize during a previous study period at the same site in February to March 2018, where we had yet collected acoustic and behavioral data, but no acoustic camera data [15,67]. The recording contexts included four settings: separation-reunion experiments at the corrals; vocalizations on the handler’s verbal cue; the elephants bathing at the river; and opportunistic observational recordings between call solicitations.

In the separation-reunion experiment, the focal individual stayed in the corral while the handler led the other elephant away, that was affiliated and housed with the focal elephant, using food and verbal cues, to a spot out of sight for the focal individual (approximate distance 100–200 m) behind dense vegetation, houses and/or straw bales. The elephants from the neighboring corrals were also moved out of sight of the focal before the start of the experiment. To initially distract and calm the focal elephant, the handler gave her three items of food (elephant “sandwiches” composed of straw and grains). After a few minutes, the focal elephant usually started contact calling. The other elephant was allowed to return by itself at any time. This resulted in a greeting ceremony where, upon reunion, the elephants vocalize in overlapping calls, come into close contact and touch and sniff each other repeatedly on different body parts (e.g., mouth, genitals, temporal glands), along with other behaviors, such as spreading and flapping ears, bouncing the trunk on the floor, raising tails and urinating [14,23,68]. Since the elephants were not equally motivated to participate in the experiments, i.e., they could not be separated or did not contact call or return to greet on their own, not every subject successfully acted as a focal, and the call types are therefore not balanced across contexts and individuals. The separations from their group mates were not part of any routine at the study site and the elephants were not used to or trained to be separated. One individual (Sunder) had an impaired hind leg due to a previous injury and we did not directly conduct the experiment with her. She was part of the group of three and was led out of sight before the actual experiment. We opportunistically recorded her calls when she reunited with the group and rated those as greeting calls. She also stayed behind at the corral when the other two elephants she was housed with were taken out for walks, and we recorded her calls as contact calls.

In the handling setting, the elephant groups were brought together in front of the camera and the handlers gave the verbal cue “bol” (speak in Nepali). The elephants are not trained to utter specific calls upon hearing this cue but start vocalizing and exhibit behaviors that are also typical for arousal situations and greeting, such as the frequent mutual touches with the trunk [14,68,69]. At the riverside, the elephants again vocalize and exhibit the greeting and arousal behaviors described above when the handlers descend after riding them to the river, which we coded as arousal, and when the elephants were reunited at the beach after bathing apart, which we coded as greeting. Along with the behavioral context, we also noted the detailed behavior (e.g., trunk touches), distance from the partner, distance from the microphone, and the mouth opening defined by visual mandible lowering (closed when no mandible lowering visible; slightly open up to an angle around 15°; wide open about 20–40°, or chewing when the mandible was moved up and down repeatedly).

### 2.4. Acoustic Camera Analysis

The acoustic camera recordings were analyzed in Noise Image (4.12.0). To locate the dominant sound source, the sound pressure level (SPL) was displayed by color coding and the resulting acoustic map projected automatically onto the optical image, where the dominant sound source appears as a color–graded sphere. The effective sound pressure at point x on the image plain was calculated by a delay–and–sum beamforming algorithm. The algorithm takes into account the sum of the relative time delays, or the phase shift when analyzed from the frequency domain. It considers each microphone position and compensates the run time or phase shift of the sound arriving at the microphone array (for details see [23]). The ranges within the time and frequency domain can be manually selected in a modifiable rectangular selection window from the waveform or spectrogram to display the acoustic map in the corresponding 2D acoustic photo. We either selected the sound from its fundamental frequency and its visible harmonics from the spectrogram to exclude background noise, specifically selected the dominant frequency contours when the calls overlapped, or specifically selected the lower or upper harmonics to identify the sound emission from multiple sources.

The algorithm assumes that monopolar uncorrelated sound sources are represented in the selected window. Several of the uncorrelated sound sources can appear as two graphic representations. When two or more correlated sound sources are present, the graphic representation of the sound source appears as one at the correct location, if its sound pressure strongly dominates the other sources. If there is a second strong source present, the round sphere of the graphic representation will be distorted in its direction. If the two sound sources are comparably strong, the graphic representation appears in-between the sources, i.e., between the trunk and mouth. By selecting only partials of the spectrogram in the time and frequency dimensions, the multiple correlated sources that occur simultaneously and or in succession can be segregated. The correct localization of the sound source, and especially the separation of the multiple sound sources, is contingent on the distance between the sources (i.e., mouth and trunk) and the distance from the source to the acoustic camera. The localizations of the sound source can be hindered by interfering background noises, strong ground reflection or multiple sound radiators. In these cases, where the sound source was not clearly localized and could not be segregated into oral or nasal parts, the sound source was termed unsure.

To measure the SPL, we only considered the calls where the whole spectrum was measurable by placing the cursor in the middle of the highest intensity color within the sound source from a 1 s selection window in the middle of the call or the whole call if its duration was less than 1 s. The videos were calculated directly from the audio file (.chl) for presentation purposes (overlap 1, framerate 25 f/s).

### 2.5. Acoustic Data Analysis

For the annotation and formant analysis of the acoustic data, we used STx (Austrian Academy of Sciences, version 4.4.6 [70]). We calculated the spectrograms (Kaiser–Bessel, bandwidth 5 Hz, window length 52 ms, frequency window: 0–1 kHz for nasal, 0–4 kHz for oral and mixed, 0–12 kHz for roars), marked the single calls and extracted their duration. We manually tracked the fundamental frequency contour from the spectrogram (rumbles: frequency 0–0.3 kHz, frame size 400–500 ms, frame increment 20 ms, frequency resolution 0.2 Hz; roars: frequency 0–0.6 kHz, frame size 200 ms, frame increment 10 ms, frequency resolution 0.8 Hz) using a custom–designed tool in Matlab [71] (The MathWorks, version R2017b), and automatically extracted the mean F0 across the call and the frequency variability index (FVI, for the parameter descriptions, see Table 2). In the roars that were both chaotic, i.e., showed high amounts of deterministic chaos, and partially tonal, we tracked the F0 where possible. We measured the spectral centroid frequency, quartiles and Wiener entropy in Rstudio (Version 1.0.153 [72]), using the analyze function of the package, soundgen [73].

To analyze the formant frequencies, we first down–sampled the sound files to 2 kHz for the nasal rumbles and 8 kHz for the mixed and oral rumbles in PRAAT, we did not down–sample the roars. We then used Stx to calculate the spectra with a Linear Predictive Coding (LPC) function over a 0.5 s time window, either directly in the middle of the call or at another location when the formants were more clearly visible, to avoid the background noise or overlapping calls. We looked at frequencies of 0–1 kHz and a LPC function coefficient of 16–20 for the nasal rumbles, 0–4 kHz and 80–100 for the oral and mixed rumbles and 0–10 kHz and 250–270 for the roars (bandwidth 1 Hz, overlap 75%). The LPC coefficients (filter order) are commonly chosen, based on the number of the expected formants 2 + 2*N (formants) [33]. The formant center frequencies can be estimated using the model of a uniform linear tube open at one end (the mouth) and closed at the other (vocal folds) and applying the equation $Fn=\left(2n-1\right)\left(\frac{c}{4VTL}\right)$, where *n* is the number of formants, *c* is the speed of sound in air (350m/s) and VTL is the vocal tract length [1]. The African elephant’s mean oral and nasal VTL is 0.75 m and 2.5 m, respectively [19,74]. In the Asian elephants, no measurements of the VTL have been published so far. However, we assumed a comparable range of the formant frequencies, due to the physical similarities of the species, and chose LPC coefficients based on our previous experience with analyzing the formants of African elephant rumbles [59]. We adjusted the coefficients when the function did not capture the spectral peak concentrations that resembled the formants from a visual inspection of the spectrum and spectrogram. This adjustment included the less prominent formants [34], and those formants that did not comply with a uniform increment across the calls. We took this conservative approach to measuring the formant positions because this is the first study to describe multiple formants in Asian elephants, thus, there are no reference values, and because we expected a deviation in the measured formant positions from those calculated based on the linear tube model, given our discovery of the mixed oral and nasal rumbles.

We determined the formant spacing (ΔF) in each call by calculating the regression slope of the measured center formant frequency positions against the (2i − 1)/2 increments of the formant spacing, as predicted by the vocal tract model [38], using the lm function (package lme4) in Rstudio (Version 1.0.153). The predicted VTL for each call was calculated using the equation $VTL=\frac{c}{2\left(\Delta F\right)}$ and their means (+/−) SD calculated per call type.

**Table 2:** Acoustic parameters measured and their description.

Acoustic Parameter	Description
Duration in s	Time from the onset until the end of the vocalization measured from the spectrogram.
Mean Fundamental Frequency (F0)	Mean of fundamental frequency values of 60 points spaced evenly across the tracked contour.
Frequency Variability Index (FVI) [75]	Calculated variable that represents the magnitude of frequency modulation across a call computed by dividing the variance in frequency by the square of the average frequency of a call and then multiplying the value by 10.
Inflection Factor (IF)	Percentage of points along the fundamental frequency’s contour showing a reversal in slope.
Jitter Factor (JF) [75]	Calculated variable that represents a weighted measure of the amount of frequency modulation by calculating the sum of the absolute value of the difference between two sequential frequencies divided by the mean frequency. The sum result is then divided by the total number of points measured minus 1 and the final value is obtained by multiplying it by 100.
Dominant Frequency (DF) in Hz	Frequency with the highest amplitude peak in the power spectrum.
Wiener Entropy	Measurement of tonality defined as the ratio of a power spectrum’s geometric mean to its arithmetic mean, expressed on a log scale. Lower values relate to higher tonality.
Quartile 25 (Q25)	Parameter characterizing the spectral energy distribution, i.e., the frequency value where 25% of the total energy is located below this value.
Quartile 50 (Q50)	Parameter characterizing the spectral energy distribution, i.e., the frequency value where 50% of the total energy is located below this value.
Quartile 75 (Q70)	Parameter characterizing the spectral energy distribution, i.e., the frequency value where 75% of the total energy is located below this value.
Spectral Centroid Frequency (SCF)	Weighted average frequency where the weights are the normalized energy of each frequency component.

### 2.6. Statistical Analysis

Statistical analyses were conducted in Rstudio. We visually inspected the variable distribution in combined boxplot-violin plots and used the Shapiro–Wilks Test to test for normal distribution. Given that most of the variables were not normally distributed, we report the median along with the mean and standard variation. The same individuals contributed calls unequally to the call type groups, resulting in a non–independent and unbalanced dataset that did not allow for controlling for individuality. Hence, we did not test the single variables for significant differences among the call types. To test for the call type discriminability based on the combined acoustic variables, we first tested for correlations among the variables, and selected the variables that did not corelate strongly with any other variable or one representative variable of a group of strongly correlating (r > 0.86) variables. We ran discriminant function analyses with leave–one–out cross classification on the log and z–transformed variables F0, duration, FVI, formant spacing (ΔF1–F10), SPL, Wiener entropy and spectral centroid frequency (SCF). We randomly selected 16 calls per call type while maintaining the maximum sample size of individuals. Since the assumption of homogeneity of variance–covariance matrices between the test groups was not met, we applied quadratic discriminant analysis [76].

## 3. Results

### 3.1. Sound Emission

**Figure 2:**
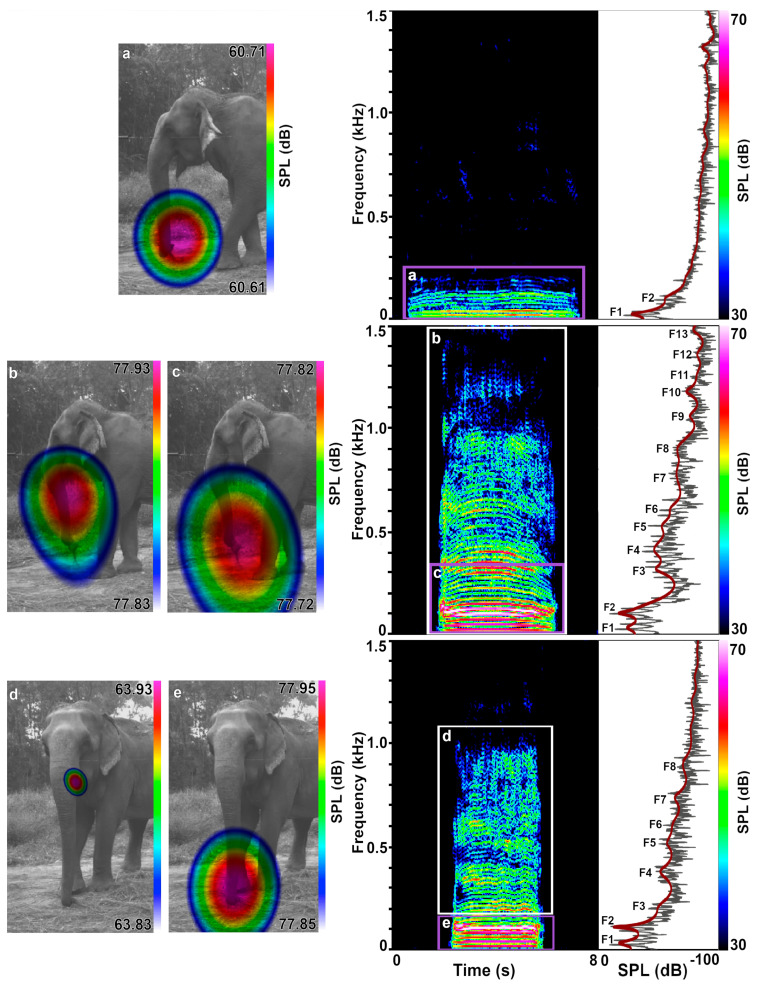
Examples of rumble emission types. Rumbles emitted in succession by Pawan (f, 55y) during the separation phase of the separation–reunion experiment showing the acoustic camera images (left), spectrograms (middle) and power spectra (right). The LPC function lines in red indicate the formant positions (F1–F13). (**a**) Nasal rumble; (**b**) Mixed emission, with oral emission but with a distorted sound sphere appearing when the whole call is selected; (**c**) Nasal part of the mixed rumble when lower harmonics are selected; (**d**) Oral part of a mixed rumble when only upper harmonics are selected; with (**e**) a more dominant nasal part when only the lower harmonics are selected. Note that during (**a**) nasal rumble emission the elephant was chewing and the mouth at times wide open as defined by jaw lowering.

**Figure 3:**
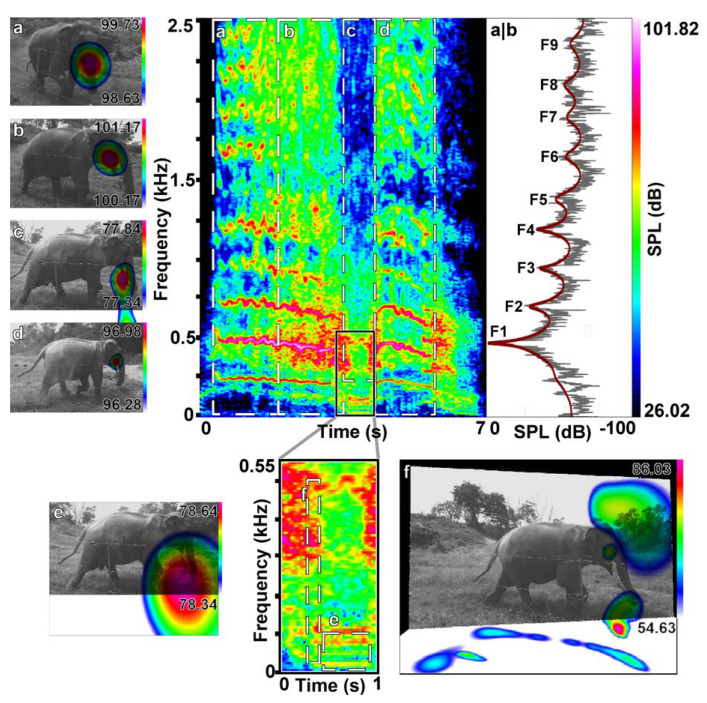
Example of a roar–rumble combination. Emitted during the separation phase of a separation–reunion experiment by Hira (f, 45y). This shows acoustic camera images of different time and frequency ranges (**a**–**f**) and the corresponding spectrogram, with a zoom–in on the rumble part (**e**,**f**) and the power spectrum (**a**,**b**) taken in the middle of the roar between selection window a and b. The LPC function line in red indicates the formant positions (F1–F9); (**a**,**b**,**d**) show the oral only emission of the roar parts; (**c**) shows the partial oral emission of the mixed emission rumble part, exhibiting a distorted sphere and the (**e**) nasal emission of the lower harmonics; (**f**) shows a pattern of simultaneous sound radiation over the front, the mouth and trunk, as well as a strong ground reflection here at the transition between roar and rumble.

In total, we captured 309 laryngeal calls with the acoustic camera. In 106 calls, the sound source was not clearly defined. Of the 203 laryngeal calls in which the sound source was identified, we recorded 189 rumbles and 14 roars. A total of 120 of 189 (64%) rumbles were emitted nasally, and 42 (22%) orally. We found that five of the orally emitted rumbles began nasally for about one second before transitioning to oral sound emission; thus, we classified them as oral rumbles and analyzed only the oral component acoustically. We classified a total of 27 (14%) calls with simultaneous oral and nasal emission (Figure 2b–e; Video S1, Supplementary Materials); six of them were part of a roar–rumble combination (Figure 3; Video S2, Supplementary Materials). We first discovered mixed oral and nasal emissions in rumbles where the sound source was not clearly detected at the mouth or the trunk tip but in–between, or where the sound sphere appeared not round but distorted, thereby indicating two sources. Here, we selected the upper harmonics only, and consequently found the sound source to be located at the mouth. The sound source appeared to be in–between the trunk and mouth or distorted again when we dragged the selection windows lower, to about 200 to 500 Hz. When selecting the lower harmonics only up to 200 to 500 Hz, the nasal sound emission dominated. We reanalyzed the rumbles that we had initially classified as nasal when we selected the spectrogram with all of the visible harmonics but exhibiting acoustic energy beyond 200 to 500 Hz. This revealed mixed emissions in these rumbles, too. All of the 14 roars were dominantly orally emitted and were part of a roar rumble call combination (Figure 3). However, when selecting the narrower frequency windows in two roar rumble combinations, we also detected evidence for radiation from the front (30 ms, 50–450 Hz, see Figure 3f and Video S3, Supplementary Materials, and 575 ms, 400–970 Hz in the second image see Supplementary Materials Figure S1), where the nostril openings are situated (Figure 1). Here as well as in several rumbles and roars we also detected ground reflections suggesting more complex patterns of sound radiation which, however, go beyond the scope of this study.

Table 3 reports the behavioral context of the call types. Most of the calls (161) were recorded in the context of contact and greeting, with 121 of those resulting from the separation–reunion experiments. The oral, nasal, and mixed calls occurred in both of the contexts. Since we did not balance the data recording across individuals nor contexts, nor corrected them for sample effort, we did not further quantify the contextual call type occurrence. The opening of the mouth defined by the lowering of the jaw was not a reliable indicator of the call emission because the nasal rumbles were also emitted with open mouths and the mixed (oral and nasal) and oral emission of rumbles were recorded with a seemingly closed mouth (Figure 2 and Figure 3; Table 4, Video S1, Supplementary Materials). Only during roar production was the mouth always visible to be slightly or widely open (Figure 3).

### 3.2. Acoustic Data Analysis

The nasal rumbles differed from the rumbles, with oral emission components in the spectral acoustic variables being concentrated in lower frequency ranges and exhibiting a lower SPL. Nonetheless, the distribution of the F0 and related parameters, tonality and duration overlapped (Table 5). The nasal rumbles had a lower SPL (51.57 ± 5.05 dB) than the mixed oral and nasal (73.5 ± 11.28 dB) and oral rumbles (62.16 ± 11.52 dB). The F0 was slightly lower in the nasal rumbles (15.594 ± 3.634) and the oral rumbles (14.98 ± 3.87 Hz) than in the mixed rumbles (22.39 ± 3.37 Hz). The mean FVI was similar in the nasal (0.07 ± 0.05) and oral (0.07 ± 0.07) and mixed rumbles (0.12 ± 0.07), as was the tonality measured as Wiener entropy (nasal: 0.23 ± 0.13, oral: 0.23 ± 0.09, mixed: 0.25 ± 0.11). The duration was similar in the nasal (5.93 ± 3.42 s) and mixed rumbles (4.12 ± 1.74 s) and lower compared to the oral rumbles (7.75 ± 3.86 s), but with overlapping variance. All of the rumble types differed clearly from the orally emitted roars in all of the measured parameters, with roars exhibiting the highest F0 (170.93 ± 45.41 Hz), SPL (88.7 ± 5.87 dB) and Wiener entropy (0.45 ± 0.13), but the shortest duration (1.42 ± 0.79 s). The frequency modulation parameters (FVI, JF, IF) were not considered in the roars because we could not consistently measure the fundamental frequency contour across the whole call in all of the calls.

The spectral acoustic energy distribution clearly differed. The spectral centroid frequency was lowest in the nasal (121.29 ± 79.97 Hz) compared to the oral (157.93 ± 58.24 Hz) and mixed rumbles (149.83 ± 44.92 Hz) and highest in the roars (417.90 ± 95.06 Hz), as were the Quartiles 25, 50 and 75 (Table 5). The dominant frequency was lowest and less variable in the nasal (16.22 ± 3.55 Hz) compared to the oral (25.64 ± 23.51 Hz) and mixed rumbles (69.99 ± 43.61 Hz) and highest in the roars (505.52 ± 392.27 Hz). The first two formants’ positions were lower and less variable in the nasal (F1 = 21.12 Hz ± 2.71, F2 = 103.34 ± 8.96 Hz) than the mixed rumbles (F1 = 33.51 ± 4.48 Hz, F2 = 110.03 ± 6.82 Hz), but not compared to the oral rumbles (F1 = 28.39 ± 6.58, F2 = 101.26 ± 14.12 Hz). The formant values in the roars were highest (F1 = 188.38 ± 89.26 Hz, F2 = 438.91 ± 99.84 Hz).

**Figure 4:**
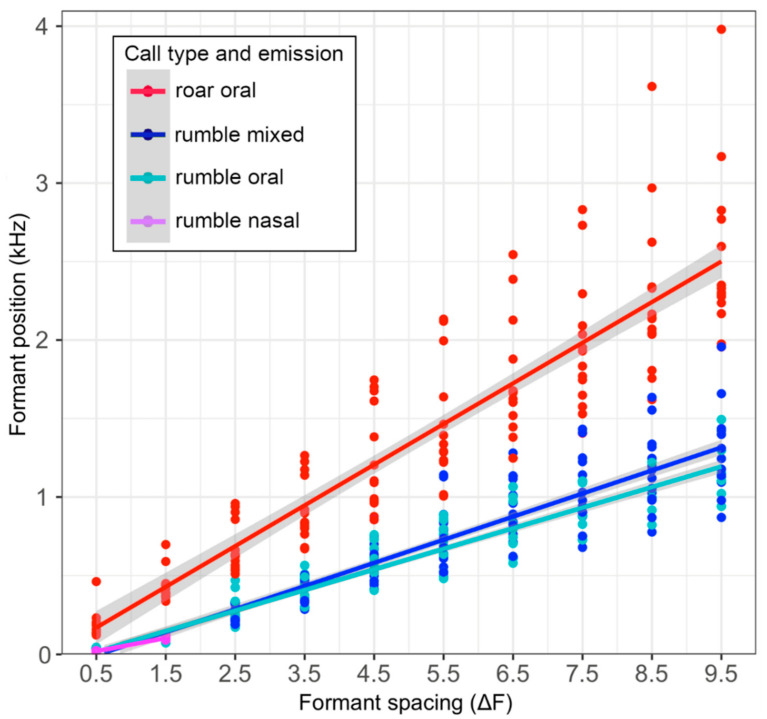
Formant position values in Hz per call and emission type plotted against the formant increment. The regression line gives the values for the formant spacing (ΔF1–F10) that is used to calculate the vocal tract length. Formant position values varied more among calls in higher formants.

The most striking difference was found in the number of the measurable formants. They never reached above two in the nasal but up to ten or more in the rumbles with oral components, and up to twenty or more and a maximum frequency bandwidth of up to 13 kHz in the roars (Figure 2 and Figure 3), with higher formant positions exhibiting considerable variance (Table 5; Figure 4). Accordingly, the formant distribution was measured between the first two formants only in the nasal rumbles (ΔF1–F2 = 81.11 ± 9.42 Hz) and translated into a nasal VTL of 218.76 ± 26.99 cm, which fits into the expected values compared to the female African elephants. Interestingly, these values were comparable to the ones of the oral (ΔF1–F2 70.73 ± 12.91 Hz and VTL of 255.57 ± 47.83 cm) and mixed rumbles (ΔF1–F2 76.66 ± 4.56 Hz and VTL of 229.08 ± 14.26 cm). This confirmed the results of the acoustic camera images showing the dominant emission of the lower frequencies through the nasal vocal tract. The calculated VTL in the roars was around 70 cm, regardless of whether the first two (73.11 ± 14.92 cm), the 3rd to 10th (70.77 ± 15.7 cm) or the 1st to 10th formants (70.88 ± 14.27 cm) were considered, and thus comparable to the VTL measured in the African elephants. When calculating the formant spacing from the 3rd formant to up to the 10th formant or across all of the formants measured, the vocal tract length in the oral (VTL ΔF3–F10 =126.02 ± 26.19 cm and VTL ΔF1–F10 = 151.46 ± 58.11 cm) and the mixed rumbles (VTL ΔF3–F10 = 119.46 ± 36.13 cm and VTL ΔF1–F10 = 126.6 ± 38.13 cm) was in-between the values for the nasal rumbles and roars, reflecting the unequal spacing between the formants.

In the spectra of the mixed and oral rumbles, we frequently observed a drop in energy (dB levels of amplitude peaks) around 200 to 300 Hz after the first two to three formants and an energy plateau around 500 to 700 Hz or 1000 to 1200 Hz. On top of this plateau, the spectral peaks occurred with a spacing similarly even to the spacing between the first two formants (see power spectra, Figure 2). This points to interactive subtractive and additive effects of the oral and nasal resonances.

The discriminate function–analysis testing call–classification within the rumble emission types, based on the parameters F0, FVI, duration, sound pressure level, ΔF1–F10, Wiener entropy and spectral centroid frequency, yielded a cross classification success of 77.08% (chance level 0.33%).

## 4. Discussion

Deploying an acoustic camera to visualize sound emissions and selecting specific frequency ranges from the spectrogram to segregate the sound sources demonstrated that adult female Asian elephants not only emit nasal rumbles, oral rumbles, and oral roars, but also rumbles that are simultaneously emitted through the mouth and trunk. This is, to our knowledge, the first study to conclusively demonstrate the coupling of oral and nasal cavities in a non-human animal. The spectral features of the nasal rumbles differed strongly from the mixed and oral calls in their acoustic energy concentration in lower harmonics but overlapped in the features of the fundamental frequency, modulation, tonality and call duration. The nasal rumbles exhibited only two formants and overall lower sound pressure levels. The lower two formants in the mixed and oral–only rumbles were in the same frequency range as in the nasal rumbles. This confirms the contribution of nasal resonances to the mixed rumbles and suggests them in the oral–only rumbles, too, although we did not detect nasal emission on the acoustic camera image. The estimated vocal tract length from the formant positions matched expected values when compared to the female African elephants for purely nasal rumbles (2.0 to 2.5 m) and oral roars (about 0.7 m) [19,23,74]. The nasal vocal tract could also be estimated from the first two formants in the mixed rumbles with oral contributions, whereas the dispersion between all or only the upper formants did not yield meaningful vocal tract estimates because of the coupled oral and nasal resonances. While the rumble emission types could be successfully classified by discriminant analysis (77%), the acoustic parameters varied considerably among and within individuals. We demonstrated that, by alternating between the oral and nasal call emission and a coupling of both, the Asian elephants increase the acoustic variability in their most frequently used call type, the rumble. This enlarges the parameter space available for biological information coding. How the Asian elephants perceive and use this flexibility to encode information remains to be investigated.

### 4.1. Velopharyngeal Coupling and Complex Formant Patterns

Our findings demonstrate that velopharyngeal coupling is not unique to humans and that its acoustic effects of enhancing, dampening and shifting the positions of spectral peaks as compared to purely oral or nasal calls in Asian elephants and humans are similar. The nasal rumbles exhibit only two lower formants, with the first around 20–30 Hz, the purely oral roars up to 20 evenly spaced higher formants, with the first formant around 200 Hz. In contrast, the mixed rumbles have both lower “nasal formants” and higher “oral formants” that are unequally spaced. Similar to human vowel production, where the oral emission is normal and thus represents the reference setting (only nasal consonants are nasally produced), adding nasal resonances leads to an overall annihilation of the spectral energy and to both the additive and subtractive effects in the relative spectral energy composition [24,28,29,36]. These effects result in additional nasal formants (poles) or antiformants (zeros), and thus relative shifts of the formant positions, reducing the formant amplitude and widening formant bandwidths for nasalized versus oral vowels. Both of the vowel types can be further quantified by measuring the relative amplitude of the peak harmonics in the formants and the spectral tilt, that is the slope of decrease in energy in the upper harmonics [28]. Applying these more detailed measurements to the elephant calls in future studies may help to better quantify the observed variability in the oral, nasal and mixed calls. The visual inspection of the “oralized” rumbles (since nasal emission is more common in elephants) with spectral energy valleys and plateaus and non-uniform formant spacing suggests spectral commonalties with the “nasalized” vowels in humans [28]. This formant structure also matches the theoretical models for adding a side branch to a linear tube vocal tract [77]. It further agrees with the potentially nasalized groans modelled for male fallow deer after CT scans of their oral and nasal cavities, where an extended gap between the third and fourth formant was described [78].

These commonalities further support the hypothesis that mammals beyond fallow deer apply velopharyngeal coupling in their vocalizations. This mechanism might be further distributed in the animal kingdom, where non-uniform formant dispersion is frequently described, for example, in ungulates [40]. The lack in data may reflect an oversight due to the complex nature of these mixed vocalizations, and methodological difficulties to identify, analyze and conclusively demonstrate their sound emission and resonances. Velopharyngeal coupling had also been suggested based on relatively lowered formant positions and an amplitude in the calls of Diana monkeys [33], rhesus macaques [32] and elephant seals [34]. In the latter, the amplitude of the first “nasal” formant was also considerably lower than the consecutive “oral” formants, and the nasal contribution in the seals, with their largely elongated and inflated vestibulum during calling, seems convincing. Nonetheless, the studies in humans using physiological and imaging techniques visualizing the velum and/or oral articulators’ position and/or nasal airflow showed that, in general, acoustic data alone cannot conclusively prove oral and nasal coupling. This is because changes in the setting of the vocal tract articulators (e.g., oral or nasal cavity constriction, tongue, lips, mouth aperture) can lead to similar acoustic effects on spectral structure [28,29,36]. Disentangling the influences of the oral or nasal resonances and the oral vocal tract configuration has proven difficult even in these human studies, where, compared to the animal models, the physiological measurements and sample sizes can far more easily be applied and the change of meaning in vocalizations is known. A considerable variation of motor settings achieved the same vocal output across the human subjects, and in the vocal output itself, the acoustic parameters differed within and between subjects. In the elephant study here, we observed a comparably large variation in the dominant frequencies and formant positions that could be attributed to different degrees of velum aperture. However, idiosyncratic morphology, or vocal strategies and articulator configuration, might also cause these effects and are aspects that require further investigation.

Contrary to the orally and nasally mixed emissions, purely oral or nasal emissions were shown in several animals. Their acoustic characteristics are concordant with our results, where the longer nasal tract leads to lower formants’ values and spacing and lower overall sound energy. The lower sound pressure levels and the spectral energy bandwidth of nasal rumbles versus those with oral contribution and the loudest, clearly oral roars, are consistent with the absorption of acoustic energy by the increased surface area and soft mucus-layered tissues within the nasal cavity and sinuses described in nasalized vowels in humans [79] and other animals’ soft nasal (e.g., dog whines or pig grunts) but loud oral calls (e.g., dog bark and pig scream) [27]. The lower formants and concentration of energy in the lower harmonics have further been shown in the nasal calls of sheep [42], goitered gazelles [39], Saiga antelopes [40] and red deer [41], and in the previous study using the acoustic camera on African elephants [23]. Interestingly, trumpets are clearly uttered through the nose but are comparably as loud as the roars and exhibit high energy in upper harmonics (Beeck, unpublished data, [15,16]). This was suggested to be caused by the alternative sound production mechanisms, including the formation of a shockwave [80].

### 4.2. Rumble Sound Production Mechanisms in African and Asian Elephants

In the African elephants, only oral and nasal emissions with significantly different formant positions but no mixed emissions were detected in the three females, so far. The two juvenile males investigated in the previous study did not utter oral calls, however, the male elephants have been observed to vocalize while the mouth is wide open in bonding contexts, apparently emitting rumbles orally (Stoeger, pers. observation). Only the first two formant positions were considered and the formant values of oral rumbles (F1 = 169.21 ± 25.61 Hz and F2 = 415.20 ± 47.71 Hz) in the African elephants match the values we observed in the oral roars of the Asian elephant females in this present study. The nasal rumbles’ formant values (F2 = 39.79 ± 5.78 Hz and F2 = 128.76 ± 32.57 Hz in the African elephants match the values we observed in the nasal, oral and mixed rumbles in the Asian elephants. As noted above, this indicates that the rumbles we classified as oral based on the acoustic image were also in fact mixed rumbles. The nasal emission may not have shown in the acoustic image because its energy might have been considerably lower than that of the oral part. The other potential explanations are that the elephant might not have been positioned in an optimal angle towards the camera, or that the background noise levels may have affected the resolution of the multiple sources. Similarly, a mixed emission in the African elephants might have been overseen due to the different methodological approaches. As opposed to the current study, where frequency ranges were selected from the spectrogram, the previous study on African elephants in 2012 [23] calculated the acoustic image directly from the waveform. This approach did not allow for the separation of the correlated sound sources, and those calls in which the sound emission was unclear were excluded from the analysis. In addition, the LPC function coefficients assume resonances of a linear tube [33]. In the mixed emission calls, this means that the coefficient selection is more subjective [28]. The different values chosen in the previous study might not have picked up the nasal formants if they were considerably lower in amplitude. In theory, we can assume that the Asian elephants are capable of producing oral rumbles with little or no nasal resonance. In turn, there are no anatomical indications that the African elephants are incapable of emitting mixed rumbles, although the frequency and context of the usage might well differ among the species. Note, however, that only the Asian elephants produce the high-pitched squeaks; we suggested by vibrating their lips [67]. Accordingly, the species differences in sound production cannot be generally excluded.

The acoustic properties of the nasal and “oralized” rumbles described here match the previous distinction of the Asian elephants low-frequency vocalization. De Silva, 2010 [65], described “growls”, as a soft low-frequency vocalization that lacks energy above 500 Hz, and is uttered with depressed cheeks and an almost closed mouth. The rumbles, in turn, have a higher spectral energy, fundamental frequencies, and variation and are uttered with the mouth open in generally more agitated contexts. We showed that mouth opening, as defined by the lowering of the jaw alone, is not a reliable indicator of sound emission. This is because the oral rumbles were also emitted through a seemingly closed (probably only very sightly opened) mouth, while the nasal or mixed rumbles were also emitted with the mouth wide open. This can be explained by the Asian elephant’s ability to tightly close the mouth in the longitudinal direction, since the upper lip is fused into the trunk [67]. This fits with the observation of the depressed cheeks when producing “growls” by de Silva. The more frequent occurrence of “growls” (60%), reported by de Silva, matches our result of 64% nasal rumbles. We expect the nasal rumbles to be produced with the larynx in its resting position—less effortful and allowing the elephant to continue feeding while vocalizing [27]. Our observation that the nasal rumbles are often produced whilst the elephant was visibly chewing (Table 3) supports this notion. In most mammals, the larynx is positioned within the nasopharynx in its resting position during breathing, and is actively retracted during loud calls [27]. While the larynx movement has not yet been studied in living elephants, the unique divided hyoid apparatus of elephants suggests great flexibility and potential for retraction [81]. The energy conservation might be enhanced by the properties of the hydrostatic long and narrow tubes that form the nasal passages in the trunk: an acoustic standing might well be induced during the nasal vocalization [82]. This may result in source filter interactions in which the higher impedance of the nasal compared to the oral pathway is more efficient in feeding power back to the source [83], a hypothesis that requires further research.

In contrast to the spectral similarities between our results and de Silva’s earlier description of the rumbles and growls, she reported differences in the fundamental frequency values and modulation, which in our study overlapped between the nasal, oral or mixed rumbles. This might reflect the fact that she recorded rumbles mainly during agitated situations and visually classified the rumble types. Similarly, the fundamental frequency differed significantly between the oral and nasal rumbles in the African elephant study [23], but both of the studies might have missed the rumbles of mixed emission that bring intermediate values and graded variance to the dataset. From our visual inspection of the rumble types, we can confirm that the more modulated and higher-pitched rumbles occur during agitated greeting. In the future, testing for arousal and behavioral context along with individual variation should help to explain more of the overlapping differences in the acoustic parameters that we observed among the emission types in this study.

Importantly, the discriminant function analysis including the overlapping parameters (fundamental frequency and variability, duration, Wiener entropy) and the differing parameters (formant dispersion, spectral centroid frequencies) still successfully classified the rumbles based on their emission, with a success rate of 77%, high above chance level (0.33%). We were unable, however, to control for individual variation and although different individuals contributed calls to each emission group, this might have led to an over classification. We assume that the variations caused by individually distinct morphology traits of the vocal tract are less prominent than the differences of the oral, nasal or mixed vocal tract resonances across individuals. Nonetheless, the classification results should be considered with caution and replicated with a sample size that allows for the testing of, and control for, individual variation in the rumble emission types.

### 4.3. Potential Functions of the Various Rumble-Production Types

We suggest that the graded variation in the acoustic parameters from the nasal, oral and mixed laryngeal calls in elephants amplifies the potential for information coding and/or long-distance communication. In the African elephants, the relative position of the first formants encodes relevant information—female estrus rumbles differentiate from non-estrus rumbles by lower first formant frequencies, higher first formant amplitudes and lower fundamental frequencies [60]. The codominant females lowered the second formant, decreasing formant dispersion in the dominance interactions compared to the rumbles produced in the low-affect contexts [84]. The alarm call warning regarding bees is distinguished by an upward shift of the first formant [85], whereas the alarm call warning regarding potential human predators is characterized by an upwards shift of the first and second formant, along with a rise in the fundamental frequencies and greater variability in both of the alarm calls compared to control rumbles [52]. How this spectral variability relates to the active modification of the oral or nasal vocal tract to encode information, and to what extent this variability relates to the physiological effects of heightened arousal remains unclear. Across mammals, the louder calls are in general oral and thus may simply reflect and transmit heightened affect. The “oralized” rumbles described here exhibit significantly more energy (around 1000 Hz and above up to 13 kHz), which matches the maximum hearing sensitivity and the maximal detectable frequency in the currently only published audiogram of a 7-year-old Asian elephant. That individual was more sensitive to low frequencies than previously tested mammals (17 Hz at 60 dB), but less sensitive to frequencies below 100 Hz than to those between 100 Hz and 5 kHz [86]. Adding the higher frequency oral vocal tract resonances to the rumbles may enable the maximal exploitation of the receiver’s attention, at least at close range. In turn, this may help the receiver in identifying or localizing the sender during the greeting ceremonies or the arousal bunching of elephants [87], where individuals join in exuberant choruses with simultaneous and overlapping calls [6], a task commonly referred to as the “cocktail party” problem [88].

Lower frequencies, however, are less prone to reverberation and absorption by vegetation and are better suited for long distance communication [89,90]. The high amplitude of the low formant positions in the rumbles may play a crucial role in the long-distance transmission of relevant information. The playback experiments have shown that the first two formants degenerate less than fundamental frequencies and higher frequencies across distances of 2.5 km in the rumbles of African female [56] and male elephants [91]. In the previous study on call emission in African elephants, the nasal sound emission of rumbles was more prominent during long-distance contact calling and oral emission during close-contact greeting [23]. By combining nasal and oral emission, elephants may be able to convey their signals over long distances while simultaneously expanding the acoustic parameter space for potential information coding over short distances.

We observed additional interesting effects in some of the acoustic images. The first is the radiation of acoustic energy from the front, where the nasal passages enter the skull, in roars and rumbles. The second is ground reflections with sometimes equal or even stronger sound pressure levels than the vocalization itself, which may play a role in sound transmission, including seismic transmission [92]. Future research must determine how the acoustic variability in elephant rumbles, caused by the flexible oral and nasal production mechanism we describe here, translates into biologically significant information. This should be coupled with specifying the role that the vocal tract configuration, including the highly flexible trunk, plays in shaping resonances.

## 5. Conclusions

Using sound imaging technology, our study is the first to conclusively demonstrate velopharyngeal coupling in a non-human animal, the Asian elephant. This adds to the previously described vocal flexibility of elephants and provides a framework for future research on the information coding and sound transmission in the poorly studied communication system of Asian elephants. We suggest that the vocal tract modifications, including the coupled contribution of oral and nasal resonances, might be more prevalent and complex formant patterns might be more relevant in mammal communication than hitherto investigated. Our findings highlight the significance of analyzing graded call variation, and those calls that do not fit into expected or predefined categories, to better understand the species-specific variability of sound production and information coding. This would be an important step forward in defining universal factors that drive the evolution of communication across species.

## Figures and Tables

**Table 1:** Subjects and call sample sizes. Number of call types combined with emission per subject; the subjects estimated age according to their handlers at the time of recording; and the subjects shoulder height as a mean value of two measurements.

			Call Types and Emission				
Subject Names	Age (Years)	Shoulder Height (m)	Rumble Nasal	Rumble Oral and Nasal	Rumble Oral	Roar Oral	Sum
Champa	41	2.40	24	9	10	10	53
Chan Chun	45	2.47	5	0	0	0	5
Dhibya	48	2.50	11	0	16	0	27
Dipendra	60	2.43	28	2	2	0	32
Hira	45	2.55	6	9	0	4	19
Pawan	55	2.41	36	4	0	0	40
Raj	42	2.49	2	1	14	0	17
Saraswati	27	2.40	1	0	0	0	1
Sunder	46	2.41	7	2	0	0	9
**Sum**			**120**	**27**	**42**	**14**	**203**

**Table 3:** Behavioral context. Number of call types combined with call emission per behavioral context.

	Rumble Nasal	Rumble Oral and Nasal	Rumble Oral	Roar Oral	Sum
Arousal	18	0	0	0	18
Command	17	2	5	0	24
Contact	26	15	6	5	52
Greeting	59	10	31	9	109
**Sum**	**120**	**27**	**42**	**14**	**203**

**Table 4:** Mouth opening defined by visual jaw lowering during call emission.

	Rumble Nasal	Rumble Oral and Nasal	Rumble Oral	Roar Oral
Closed	65	3	8	0
Slightly open	19	13	8	3
Wide open	6	9	12	9
Chewing	14	0	5	0
Unknown	16	2	9	2

**Table 5:** Results of acoustic parameters measured per call emission type. The table shows sample sizes, median and mean values ± SD.

	Rumble Nasal			Rumble Oral and Nasal			Rumble Oral			Roar Oral		
Parameters	*N*	Median	Mean ± SD	*N*	Median	Mean ± SD	*N*	Median	Mean ± SD	*N*	Median	Mean ± SD
Duration in s	120	5.25	5.93 ± 3.42	27	3.94	4.12 ± 1.74	42	7.36	7.753 ± 3.86	14	1.28	1.42 ± 0.79
SPL in dB	72	51.7	51.57 ± 5.05	24	74	73.5 ± 11.28	27	65.2	62.159 ± 11.524	12	88.7	88.7 ± 5.87
Mean F0	75	14.96	14.98 ± 3.87	21	21.78	22.39 ± 3.37	37	15.78	15.594 ± 3.634	11	172.4	170.93 ± 45.41
FVI	75	0.06	0.074 ± 0.05	21	0.12	0.12 ± 0.07	37	0.06	0.074 ± 0.068	11	0.04	-
IF	75	0.39	0.39 ± 0.11	21	0.27	0.28 ± 0.12	37	0.35	0.33 ± 0.11	11	0.17	-
JF	75	3.05	3.28 ± 1.08	21	3.16	3.27 ± 0.92	37	2.7	2.74 ± 1.09	11	2.07	-
DF in Hz	64	16.6	16.22 ± 3.55	20	97.81	69.99 ± 43.61	27	18.55	25.643 ± 23.51	14	378.29	505.52 ± 392.27
**Spectral**												
Wiener Entropy	66	0.17	0.23 ±0.13	21	0.21	0.25± 0.11	29	0.20	0.23± 0.09	15	0.47	0.45 ± 0.13
Q25 in Hz	66	13.68	20.80± 53.34	21	42.10	53.93± 28.46	29	27.36	30.93± 12.21	15	300.00	278.36± 111.92
Q50 in Hz	66	34.73	53.52± 82.40	21	92.63	102.46± 48.84	29	63.15	70.54± 27.87	15	408.42	388.93± 138.15
Q 75 in Hz	66	84.73	142.68± 129.58	21	134.73	185.67± 105.94	29	140.00	173.92± 99.84	15	510.52	529.67± 112.71
SPF in Hz	66	93.74	121.29± 79.97	21	133.44	157.93±58.24	29	148.06	149.83± 44.92	15	421.55	417.90± 95.06
**Formant**												
F1 in Hz	61	21.48	21.12 ± 2.71	19	33.2	33.51 ± 4.48	28	26.86	28.39 ± 6.58	13	157.47	188.38 ± 89.26
F2 in Hz	34	103.25	103.34 ± 8.96	18	110.84	110.03 ± 6.82	19	105.47	101.26 ± 14.12	13	405.03	438.91 ± 99.84
F3 in Hz	0	0	0	17	242.19	260.28 ± 56.47	16	240.23	259.32 ± 83.66	13	631.71	692.63 ± 161.82
F4 in Hz	0	0	0	17	437.5	408.72 ± 75.59	14	464.36	440.64 ± 79.13	13	869.04	936.07 ± 200.37
F5 in Hz	0	0	0	16	598.14	583.26 ± 125.44	13	545.9	577.96 ± 117.67	13	1097.53	1244.10 ± 335.26
F6 in Hz	0	0	0	16	750.49	755.17 ± 187.56	12	704.59	694.59 ± 144.73	13	1314.15	1467.41 ± 389.24
F7 in Hz	0	0	0	15	889.65	908.2 ± 196.67	12	814.45	823.24 ± 176.55	13	1644.11	1719.8 ± 410.19
F8 in Hz	0	0	0	14	960.94	1030.41 ± 229.54	9	878.91	899.31 ± 160.21	13	1881.59	1949.07 ± 439.4
F9 in Hz	0	0	0	14	1145.51	1162.25 ± 243.24	6	1021.6	1033.32 ± 155.65	13	2139.03	2270.22 ± 540.77
F10 in Hz	0	0	0	13	1244.14	1293.17 ± 289.66	6	1127.93	1167.91 ± 199.95	13	2315.92	2533.59 ± 555.53
ΔF1–F10 in Hz	34	-	81.11 ± 9.42	18	-	148.99 ± 40.02	19	-	127.61 ± 36.38	14	-	258.95 ± 66.65
ΔF1–F2 in Hz	34	-	81.11 ± 9.42	18	-	76.66 ± 4.56	19	-	70.73 ± 12.91	14	-	249.73 ± 56.82
ΔF–F10 in Hz	0	-	0	17	-	160.96 ± 53.85	14	-	144.93 ± 31.99	14	-	261.33 ± 71.15
**VTL**												
ΔF1–F10 in cm	34	-	218.76 ± 26.99	18	-	126.6 ± 38.13	19	-	151.46 ± 58.11	14	-	70.88 ± 14.27
ΔF1–F2 in cm	34	-	218.76 ± 26.99	18	-	229.08 ± 14.26	19	-	255.57 ± 47.83	14	-	73.11 ± 14.92
ΔF3–F10 in cm	0	-	0	17	-	119.46 ± 36.13	14	-	126.02 ± 26.19	14	-	70.77 ± 15.7

## Data Availability

The data presented in this study are available on request from the corresponding author.

## Supplementary Materials

The following supporting information can be downloaded at: https://www.mdpi.com/article/10.3390/ani12162119/s1, **Video S1: Acoustic video of rumble emission types.** The video shows the acoustic camera image and the waveform below of rumbles emitted in succession by Pawan (f, 55y) during the separation phase of the separation–reunion experiment. The rumbles emitted are the same depicted in Figure 2, with first a nasal rumble emitted while chewing, second a mixed rumble with the oral emission being dominant and third, a mixed rumble with the nasal emission being dominant. The mixed emission is visible by the colored sound sphere moving in between the mouth and trunk, whereas it remains steady at the trunk tip in the first, purely nasally emitted rumble. **Video S2: Setup of acoustic camera and acoustic video of a roar–rumble combination.** The video first shows the set-up of the acoustic camera in front of an elephant enclosure. In the following it shows Hira (f, 45y) emitting rumbles during the separation phase of a separation–reunion experiment and the acoustic camera image of the roar–rumble combination depicted in Figure 3, followed by another shot of the camera setup. **Video S3: Demonstration of SPL measurement from the acoustic image.** This video demonstrates the SPL (here Lp) measurement from the acoustic image including the radiation from the elephant’s front and a strong ground reflection, depicted also in Figure 3f. **Figure S1: Second example of a roar–rumble combination.** Emitted during the separation phase of a separation–reunion experiment by Champa (f, 41y) showing the acoustic camera images of different time and frequency ranges (a–d) and the corresponding spectrogram: (a) shows the oral only emission of the roar part; (b) the frontal sound radiation during the roar, (c) the partial oral emission of the mixed emission rumble and (d) the nasal emission of the rumble’s lower harmonics.
