# Supplementary figures and images for: Sound Visualization Demonstrates Velopharyngeal Coupling and Complex Spectral Variability in Asian Elephants
Source: Animals (Basel). 2022 Aug 18;12(16):2119. doi: 10.3390/ani12162119 (PMC9404934; doi:10.3390/ani12162119)

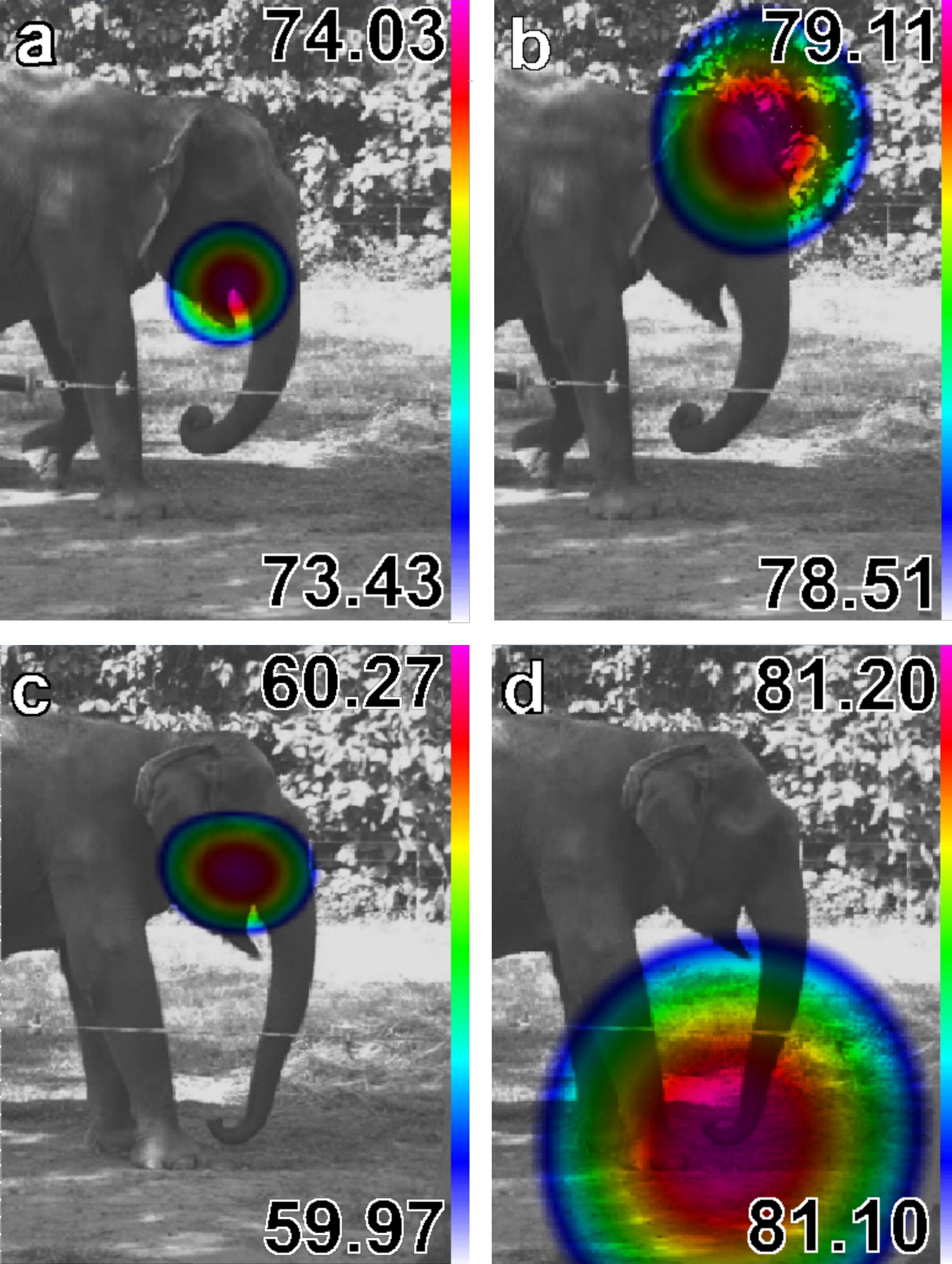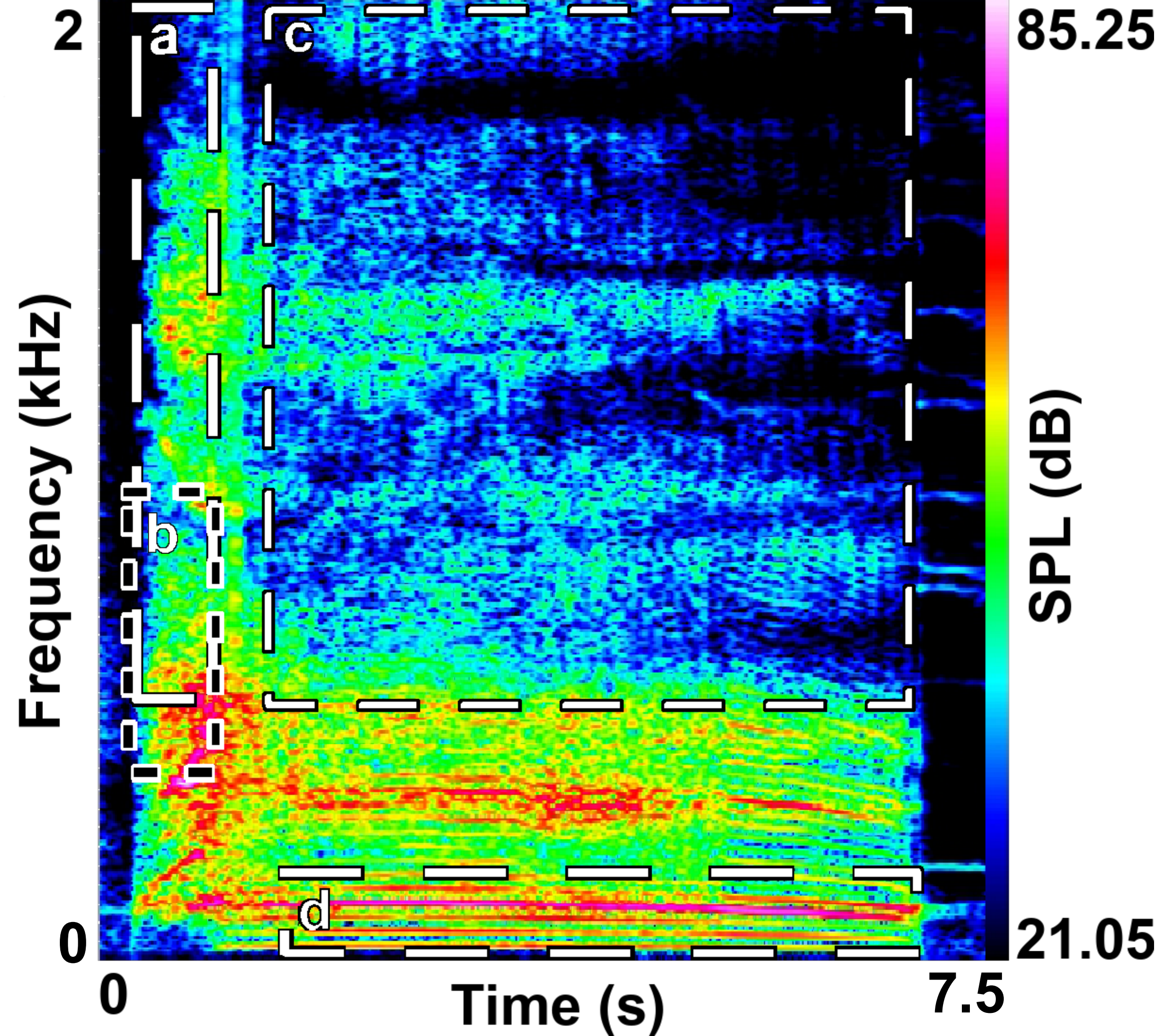

Supplement: Supplementary file 1 [file animals-12-02119-s001.zip › Figure_S1_roar.pdf]
